# A Critical Overview of Targeted Therapies for Glioblastoma

**DOI:** 10.3389/fonc.2018.00419

**Published:** 2018-10-15

**Authors:** Kewal K. Jain

**Affiliations:** Jain PharmaBiotech, Basel, Switzerland

**Keywords:** brain cancer, glioblastoma, personalized therapy, targeted delivery, malignant glioma, oncolysis, cancer immunotherapy, gene therapy

## Abstract

Over the past century, treatment of malignant tumors of the brain has remained a challenge. Refinements in neurosurgical techniques, discovery of powerful chemotherapeutic agents, advances in radiotherapy, applications of biotechnology, and improvements in methods of targeted delivery have led to some extension of length of survival of glioblastoma patients. Refinements in surgery are mentioned because most of the patients with glioblastoma undergo surgery and many of the other innovative therapies are combined with surgery. However, cure of glioblastoma has remained elusive because it requires complete destruction of the tumor. Radical surgical ablation is not possible in the brain and even a small residual tumor leads to rapid recurrence that eventually kills the patient. Blood-brain barrier (BBB) comprising brain endothelial cells lining the cerebral microvasculature, limits delivery of drugs to the brain. Even though opening of the BBB in tumor core occurs locally, BBB limits systemic chemotherapy especially at the tumor periphery, where tumor cells invade normal brain structure comprising intact BBB. Comprehensive approaches are necessary to gain maximally from promising targeted therapies. Common methods used for critical evaluation of targeted therapies for glioblastoma include: (1) novel methods for targeted delivery of chemotherapy; (2) strategies for delivery through BBB and blood-tumor barriers; (3) innovations in radiotherapy for selective destruction of tumor; (4) techniques for local destruction of tumor; (5) tumor growth inhibitors; (6) immunotherapy; and (7) cell/gene therapies. Suggestions for improvements in glioblastoma therapy include: (1) controlled targeted delivery of anticancer therapy to glioblastoma through the BBB using nanoparticles and monoclonal antibodies; (2) direct introduction of genetically modified bacteria that selectively destroy cancer cells but spare the normal brain into the remaining tumor after resection; (3) use of better animal models for preclinical testing; and (4) personalized/precision medicine approaches to therapy in clinical trials and translation into practice of neurosurgery and neurooncology. Advances in these techniques suggest optimism for the future management of glioblastoma.

## Introduction

Glioblastoma is the most lethal primary brain tumor. Since the first surgical resection of a malignant astrocytoma was done in 1884, attempts to cure primary cancer of the brain by resection have been made since the start of modern neurosurgery more than a century ago. Since my first encounter with a case of glioblastoma at the start of my neurosurgical career 60 years ago, I have seen improvements in surgical technique, radiotherapy, and chemotherapy increase the median length of survival of patients only from 6 to 15 months. While standard of care for newly diagnosed glioblastoma include maximal resection, followed by radiation therapy given concomitantly with temozolomide followed by adjuvant TMZ chemotherapy, median time to progression is 6 months and overall median survival 15 months. Almost all tumors recur with more aggressive form of tumors and there is no standard of care for recurrent GBM. During the past two decades, applications of biotechnology and several innovative approaches have been tested both in the laboratory and clinical trials; their impact on survival is negligible because glioblastoma remains incurable. Currently, most of the projects for advancing therapy for glioblastoma listed in Table 1 are focused on targeted delivery to the tumor and aim to selectively eradicate it without damaging the surrounding brain. Nanobiotechnology plays an important role in targeted delivery of therapy to glioblastoma and will be discussed in a separate section. Various innovative therapies will be critically evaluated including examples from the 1269 clinical trials listed at the US government web site^1^. To start with I will review refinements in surgery including combination with other innovations.

**Table 1:** Innovative therapies for glioblastoma.

**New chemotherapeutic agents**
**Innovations for the delivery of anticancer drugs** Intraoperative polymer implants in residual tumor bed containing anticancer drugs Magnetic cationic microsphere delivery system Stereotactic implantation of microspheres containing anticancer drugs Lipid-coated microbubbles as a delivery vehicle
**Strategies to overcome the blood-tumor barrier for delivery of chemotherapy** Intra-arterial chemotherapy Nanotechnology-based controlled delivery through blood-brain barrier
**Chemotherapy sensitization** Hyperbaric oxygen Photodynamic therapy for chemosensitization
**Innovations of radiotherapy** Boron neutron capture therapy Brachytherapy: implantation of interstitial radiation-emitting seeds into the tumor Enhancing effect of radiotherapy by hyperbaric oxygen Radiosurgery: enhancing effects of ionizing radiation
**Inhibition of tumor growth** Receptor tyrosine kinases as a signal blocker to hinder the growth of gliomas Telomerase inhibition Antiangiogenesis therapy Polyinosinic-polycytidylic acid given intramuscularly Thalidomide, systemic administration Targeting epidermal growth factor receptor-mediated metabolic pathway
**Local destruction of tumor** Genetical modified bacteria for tumor-specific lysis Hyperthermia Interleukin-4 fusion toxin injection Intraoperative photodynamic therapy Oncolysis by genetically modified viruses Tumor treating fields
**Immune therapy** Brain tumor vaccines Immune checkpoint blockade Monoclonal antibodies Radiolabeled antibodies injected directly into the tumor Recombinant interleukin-2 and lymphokine activated killer cells Recombinant immunotoxins specific for epidermal growth factor receptor
**Cell therapy** CAR-T cell therapy Encapsulated cells engineered to produce therapeutic molecules Glioma stem cell therapy Grafting of stem cells producing therapeutic molecules, such as IL-4 gene
**Gene therapy** Apoptosis-inducible FADD/MORTI gene transfer Deoxycytidine cDNA as sensitizer for cytotoxic effect of cytosine arabinoside Direct intratumoral injection of genetically modified neurotrophic viruses E. coli gpt gene delivery to sensitize glioma cells to prodrug 6-thioxanthine Insertion of drug sensitivity genes Suicide gene therapy: herpes simplex virus-thymidine kinase Viral vectors containing radiation-inducible promoters
**Gene suppression** Antisense RNAi

Although several innovations in treatment of glioblastoma have been introduced during the past three decades, evaluation of their efficacy is mostly limited to observation of progression free survival and overall survival. While CT and MRI measure tumor size, metabolic, and other changes at molecular level in response to treatment can be better indicators of response in the absence of an initial reduction of size. Conventional preclinical studies evaluating experimental drugs in cell lines *in vitro* do not recapitulate conditions of *in vivo* tumor microenvironment; even clinical trials conducted in mixed population are not adequate to realize impact of an experimental drug.

## Refinements in surgery

There have been considerable refinements in surgical techniques. In the pre-brain imaging (CT and MRI) era, preoperative diagnosis with pneumoencephalography (which showed mostly the location and mass displacement and cerebral angiography (crude vasculature patterns and avascular areas of necrosis) raised suspicion of malignancy, which had to be confirmed by histological examination. Compared to modern refinement, neurosurgery of glioblastomas 60 years ago was crude as compared to meticulous dissection of benign brain tumors because it was considered a palliative procedure to relieve intracranial pressure and prolong life for a few months with resignation to the fact that the patient was going to die.

Apart from providing adequate sample for histological analysis and removal of a mass to reduce raised intracranial pressure, excision of a tumor provides a cavity for application of local therapies for destruction of residual tumor mass and prevention of recurrence. Maximal removal that is consistent with neurological preservation is usually carried out and has been shown to prolong survival but does not reduce mortality. Radical extirpation of the tumor is often aimed at but is not possible due to infiltration of the tumor into the surrounding brain.

Refinements in brain imaging techniques have contributed considerably in improving planning of surgical procedure. Intraoperative imaging, particularly MRI and use of 5-aminolevulinic acid helps in defining the margins of glioblastoma and for maximizing the extent of resection. According to a systematic review of randomized clinical trials, the impact of image-guided surgery on survival and quality of life are uncertain (1).

Techniques such as cortical mapping, fluorescence-guided surgery, and intraoperative mass spectrometry are routinely used in the operating room for brain tumor resection. Optical coherence tomography, still in experimental stage, may fill the need for a non-invasive approach for real-time distinction between tumor and normal brain. Postoperative imaging provides a useful baseline for size of residual tumor and further evaluation of response to adjunctive therapies.

One of the major refinements in neurosurgical techniques was the introduction of operating microscope, which had a remarkable impact on improving cerebrovascular surgery. It provides better visualization of distinction between the tumor and the normal brain to avoid damage to normal structures. Other refinements in tools for removing tumor tissue include ultrasonic aspiration to minimize trauma and laser vaporization to reduce bleeding and destroy cells in tumor bed by thermal effect. The FDA-approved NeuroBlate® System (Monteris Medical) is a minimally invasive robotic laser thermotherapy tool for glioblastoma that is being studied in the prospective multicenter clinical trial #NCT02392078 due for completion in 2020. The NeuroBlate System is used with MRI to provide a real-time image of a patient's brain for guiding the surgeon. This device is meant for glioblastomas that are not suitable for routine surgery due to their location. The aim is improvement in quality of life of the patient rather than prolonging survival.

### Combination of surgery with other innovations

Surgery is supplemented with innovations in chemotherapy and radiotherapy that will be described separately. In addition to systemic chemotherapy and postoperative cranial radiotherapy, surgery provides an opportunity to apply some therapies during the procedure. Examples are implantation of chemotherapeutic agents and photodynamic therapy.

#### Implantation of wafers for local delivery of chemotherapeutic agents

Gliadel wafers consist of biodegradable polymer containing the chemotherapeutic agent BCNU (bis(bis-chloroethylnitrosourea), which are implanted during surgery at the tumor site diffuse into the surrounding tissue and provide therapeutic dose of BCNU locally. A systematic review of phase III trials has shown an increased overall survival from sequentially combining Gliadel wafers with radiotherapy and systemic temozolomide (2).

#### Photodynamic therapy

Photodynamic therapy (PDT) is based on release of singlet oxygen with toxic effects on the tumor that occurs when a photosensitizer at the tumor site is exposed to laser light of a certain wavelength. PDT for cancer using talaporfin sodium with laser is approved in Japan. Progression-free survival of 1 year and an overall survival of 2 years and 8 months was shown in an open clinical trial of intraoperative PDT in glioblastoma (3). A prospective study on patients with glioblastoma used fluorescence from talaporfin sodium exposed to laser with wavelength of 600 nm for intraoperative diagnosis of malignant glioma, which was followed by PDT at laser wavelength of 400 nm (4). According to the authors, fluorescence from malignant tissues was at least partially due to the involvement of microvascular structures. A pilot clinical trial, the INDYGO (INtraoperative photoDYnamic Therapy for GliOblastomas) is planned as an addition to the standard of care of glioblastoma as a requirement prior to a randomized clinical trial (5). In this clinical trial, PDT treatment following fluorescence-guided surgical resection will involve delivery of 5-aminolevulinic acid (ALA) processed by the cells to generate a photosensitizer protoporphyrin IX (PIX).

## Role of improvements in diagnostic technologies

Improvements in diagnostic technologies have played an important role in understanding the genetics and molecular biology of glioblastoma as a basis for developing therapeutics and evaluating their efficacy. Molecular diagnostics is used for discovery of biomarkers of brain tumors and some biomarkers can serve as diagnostics as well as targets for developing therapies, which explains the overlap between the two (6).

### Molecular diagnostics

Molecular analyses of genetic alterations in astrocytomas have been carried out to identify pathways leading to glioblastoma. Molecular diagnostics is an important basis for developing personalized therapy of glioblastomas. The most frequent single-gene alterations are mutation of the p53 tumor suppressor gene, amplification of the epidermal growth factor receptor (EGFR), homozygous deletion of the CDKN2a gene, and mutation of the PTEN (phosphatase and tensin homology) gene on chromosome chromosome 10q23. While most frequent chromosomal alterations are loss of chromosomes 10 and 9p and the gain of chromosomes 7 and 19 and were discovered by studies involving high-resolution comparative genomic hybridization (7), The assays to assess these genetic or chromosomal alterations have emerged as molecular biomarkers of glioblastoma as well.

Magnetic resonance spectroscopy (MRS) mediated detection of oncometabolite 2-hydroxyglutarate (2HG) produced in tumors harboring mutation in the isocitrate dehydrogenase1 (IDH1). A prospective imaging study showed positive correlation between 2HG concentrations tumor cellularity, which differ significantly among high- vs. low-grade gliomas (8). These data provide rationale for adding 2HG MRS into clinical practice IDH-mutated gliomas.

Cell-free DNA shed by cancer cells is a rich source of tumor-specific biomarkers, but DNA from CNS tumors cannot usually be detected in the blood. However, using patient-specific mutations as biomarkers, detectable levels of CSF tumor DNA were identified in 74% of brain tumors such as glioblastomas that abutted on CSF spaces (9).

Alterations in EGFR, PDGFRA, PTEN, TP53, NF1, CDKN2A/B, and TERT promoter mutations are commonly found in primary glioblastoma, while the prevalence of IDH mutations is high in grades II and III astrocytomas, secondary glioblastoma, oligodendrogliomas, and oligoastrocytomas (10). Survival benefit from surgical resection varies according to IDH1 genotype in glioblastomas, as better prognosis is observed in the IDH1 mutant subgroup following maximal surgical resection (11). Amplification of receptor kinases such as EGFR and PDGFRA (platelet-derived growth factor receptor alpha) are relevant to the prognosis of glioblastoma, and both may coexist in a tumor. Inhibition of both EGFR and PDGFR is essential for eliminating kinase pathway activity in glioblastomas with mixed cell types (12). In view of the cytogenetic heterogeneity of glioblastoma, stratification for prognosis should take into consideration cytogenetic alterations involving chromosomes 7, 9, and 10 which are the most frequent alterations (13).

There is a considerable interest in next-generation sequencing-based technology as it allows comprehensive mapping of genetic alterations such as single nucleotide polymorphism (SNP), fusions, and copy number variations and the epigenetic landscape of DNA methylation in brain tumors. A customized next-generation sequencing gene panel involving 0 genes commonly altered in brain tumors have been developed for the detection of Sahm et al. (14). With the emergence of numerous therapeutic targets, this approach will be important for making decisions about treatment, and classification of brain tumors based on genetic information.

There is a need for a practical method to delineate glioblastomas from adjacent normal brain tissue during surgery. Several intraoperative imaging techniques have been developed for determining the resection margin in brain tumors; these include neuronavigation, MRI, ultrasound, Raman spectroscopy, and optical fluorescence imaging. Combined with discovery of contrast agents, both MRI and optical fluorescence imaging have improved resectability of brain tumors (15). Fluorescence-guided surgery uses preoperative oral 5-aminolevulinic acid (5-ALA) for intraoperative visualization of glioblastoma tissue and enables the neurosurgeon to differentiate tumor from normal brain for achieving a more extensive resection of the tumor as compared to that possible with use of conventional operating room light (16).

### Biomarkers

There are several biomarkers of glioblastoma and those relevant to management are listed in Table 2. Some biomarkers can be used to control response to therapy, diagnosis, and prognosis.

**Table 2:** Classification of biomarkers of glioblastoma that are relevant to management.

**Cytogenetic biomarkers** BRAF mutation EGF, latrophilin and 7 transmembrane domain-containing 1 on chromosome 1 (ELTD1) EGFRvIII deletion Loss of heterozygosity (LOH) on chromosomes 1p, 19q, 17p, and 10q Loss of p16 tumor suppressor gene pathway Loss of p53 tumor suppressor gene MAGE-E1, a glioma-specific member of MAGE (melanoma-associated antigen) family Mdm2 amplification in 15% of malignant gliomas PTEN deletion or mutation RBI wild type, no mutation
**Methylation profiling of brain tumors** 06-methylguanine-DNA methyltransferase (MGMT) promoter methylation (17) Detection of methylation-dependent DNA sequence variation: methylSNP Methylation of TMS1, an intracellular signaling molecule
**Protein biomarkers** ALDH1A3 in glioma-like stem cells in glioblastoma CSF protein profiling: N-myc oncoprotein, caldesmon, attractin Receptor protein tyrosine phosphatase Serum protein fingerprinting: circulating exosomes containing mRNA, miRNA, and angiogenic proteins
**Metabolite biomarkers detected by magnetic resonance spectroscopy (MRS)** Choline Lactate N-acetylaspartate (diminished)
**MicroRNAs (miRNAs)**
**Prognostic biomarkers** 14-3-3zeta positive expression Human telomerase reverse transcriptase (hTERT) transcripts; survival is worse in high hTERT expressors Isocitrate dehydrogenase-1 (IDH1) mutation status
**Biomarkers of response to therapy** Biomarkers to predict response to EGFR inhibitors MRI as biomarker for response of brain tumor to therapy

ALDH1A3, an enzyme plays important role in alcohol metabolism and lipid peroxidation is a specific biomarker for glioma stem-like cells (GSCs), and cells with high expression of ALDH1A3 expression are shown to be highly tumorigenic (18). In samples of glioblastoma from patients, high expression levels of ALDH1A3 were associated with a more rapid fatal course than were tumors with low levels. Small molecule inhibitor of ALDH have been developed, GAI1, an ALDH inhibitor abated glioma sphere forming ability of GSCs in cell culture and the xenograft growth of glioblastoma cells. Thus, GA11 is can be promising therapy or sensitizing strategy for glioblastoma, and clinical trials to test therapeutic potential of GAI1 have been planned (18).

Promoter hypermethylation of *MGMT* (O6-methylguanine-DNA methyltransferase) gene have been associated with response to alkylating chemotherapy (19) is an established biomarker for the alkylation therapy of glioblastoma. The PredictMDx test is a commercially available epigenetic assay for testing methylation status of the *MGMT* gene and has impact on decision making especially for the patients with newly diagnosed glioblastoma (20). *MGMT* promoter methylation is also a prognostic biomarker for combination therapies such as temozolomide combined with carmustine (BCNU) wafer implantation (21) and is being tested in phase II-III clinical trials (NCT02152982) of targeted therapy involving temozolomide and PARP inhibitor veliparib (22).

Advanced data mining and a novel bioinformatics were used with associative analysis to accurately identify ELTD1 (epidermal growth factor, latrophilin, and 7 transmembrane domain-containing 1 on chromosome 1) as a biomarker of gliomas in preclinical and clinical diagnosis (23). A clinical study showed that expression of 14-3-3zeta observed in ~74.5% of patients with glioblastoma and is associated with a lower overall survival rate and median survival time than patients with no14-3-3zeta expression (24). This study shows that 14-3-3zeta positive expression is an independent prognostic biomarker of glioblastoma.

Among various biomarkers, only isocitrate dehydrogenase (IDH) mutations (prognostic), MGMT promoter methylation, and 1p/19q co-deletion (predictive) are being routinely used in clinic for glioma patients in the USA as well as the UK. More biomarkers are being tested in clinical trials, and it will be important in the future to distinguish prognostic biomarkers from predictive biomarkers to enable personalized therapeutic choices with least toxicity and better outcomes for patients with malignant gliomas (25).

Examples of biomarkers that are also targets for therapy of glioblastoma are:

- EGFRvIII amplification is targeted by EGFR vaccine rindopepimut.
- KIT amplification or mutation is target is targeted by KIT inhibitor imatinib
- PDGFRA amplification is targeted by PDGFR inhibitor dasatinib
- PTEN deletion or mutation is targeted by AKT inhibitor or mTOR inhibitor voxtalisib
- MDM2 amplification is targeted by a MDM2 inhibitor such as AMG232
- TP53 wild-type is targeted by a MDM2 inhibitor such as AMG232
- RB1wild-type is targeted by CDK4/6 inhibitor ribociclib

### MicroRNAs

MicroRNAs (miRNAs) are small (about 22 nucleotide long) non-coding RNA that regulate gene expression by preventing mRNA translation by base pairing on complimentary sequences of RNA, have been implicated in various pathological processes in the human body. Using glioblastoma tissues and cell lines several groups of miRNAs have been identified whose expression is significantly altered in glioblastoma. Dysregulation of miRNA regulated genes is considered one of the key mechanisms in pathogenesis of glioblastoma, and several miRNAs involved in tumor initiation and progression have been described (26). Therefore, miRNAs are not only excellent diagnostic biomarkers, but also serve as targets for molecular therapies. Targeting miRNA(s) could alter multiple genes simultaneously and may prove more effective than targeted focused at targeting single gene or pathway (27). miRNAs, by playing a role in glioma stem cells (GSCs), may predispose to development of resistance to temozolomide (TMZ) therapy of glioblastoma, and dysregulation of GSCs pathways by targeting miRNA could provide an effective strategy against TMZ-resistant glioblastoma (28).

Specific to glioblastoma, the plasma levels of miR-21, miR-128, and miR-342-3p are found elevated and can distinguish glioblastoma patients from healthy controls or other types of brain tumors (29). Interestingly, the plasma levels of these 3 miRNAs dropped in glioblastoma patients drop following treatment by surgery plus chemo-radiation indicating that diagnostic tests can be developed to assess disease progression or recurrence.

Pronounced reduction of miR-218 in patients with highly hypoxic and necrotic glioblastoma contributes to temozolomide resistance as demonstrated in transplanted glioblastoma in mice, whereas tumor growth is significantly reduced with increase in survival in response to temozolomide in mice with high miR-218 (30). According to these authors, miR-218 downregulates expression of certain components in receptor tyrosine kinase pathway, and hypoxia-inducible factor 2α. Understanding the molecular basis of miR-218-mediated chemoresistance would be useful for the development of targeted therapies.

Molecular-targeted therapy based on miRNA expression in cancer stem cells can facilitate personalized and effective treatment strategies for glioblastoma in the future (27).

## Innovations in chemotherapy

Although temozolomide chemotherapy is the standard treatment for newly diagnosed glioblastoma, which is generally well tolerated with lower toxicity than nitrosoureas. However, combination to temozolomide with other anticancer agents have also been investigated.

### Innovations in delivery of chemotherapy

To avoid systemic toxicity of chemotherapy, various methods have been used to limit application to the tumor such as implants in tumor cavity following surgical excision, targeted delivery to glioblastoma following systemic administration, and selective delivery of higher concentrations to the tumor, e.g., by intraarterial chemotherapy. Delivery of monoclonal antibodies (MAbs) in glioblastoma will be discussed in the following section on immune therapy.

#### Drug formulations for improved delivery to brain tumors

Delivery of anticancer agents is limited by their inability to reach therapeutic levels in brain tumors with maximally tolerated dose regimens. Drug targeting by conjugating with protein such as transferrin has been extensively studied, as a targeting molecule transferrin helps the transport of drug to glioblastoma which contains abundant transferrin receptors on the surface. Transferrin-bearing therapeutic agents can be targeted to their site of action on brain tumors (31).

Thermosensitive liposomes can encapsulate drugs to release them at the target site in the tumor in response to hyperthermia without exposing the surrounding normal brain to toxicity. Increase in efficacy of doxorubicin by release from a thermosensitive liposomal nanocarrier as compared to non-thermosensitive liposomes has been demonstrated in a mouse model of human glioblastoma (32).

Angiopep-2 (An2) for BBB transcytosis and anti-CD133 MAb for specific delivery to glioma stem cells have been incorporated in a dual-targeting immunoliposome encapsulating temozolomide (33). A significant reduction in size of implanted glioblastoma in the rat was shown after intravenous administrations of the dual targeting system, indication a potential use in treatment of patients with glioblastoma.

#### Convection-enhanced delivery

Convection-enhanced delivery (CED) involves direct delivery of a therapeutic agent to the brain by injection or a catheter propelling the agent through interstitial spaces under a pressure gradient rather than passive diffusion. It has been used for both chemotherapy drugs and for delivery of macromolecules of some biological therapies for glioblastoma.

A prospective, dose-escalation phase Ib study of the topoisomerase-I inhibitor, topotecan, given by CED in patients with recurrent glioblastoma resulted in significant efficacy as assessed by radiographic changes while using doses that were well tolerated by the normal brain (34). Overall survival was prolonged in this study with minimal drug toxicity, which helped to determine the maximum tolerated dose for further phase II/III studies.

#### Modification of BBB for delivery of chemotherapeutic agents

Several chemotherapeutic agents used for glioblastoma such as nitrosoureas, temozolomide can cross the intact BBB, but larger molecules such as MAbs may not do so. BBB permeability may be increased in glioblastoma, but this is not a reliable factor in assessing delivery of a therapeutic for brain tumors. Several strategies for drug delivery across the BBB have been described; some of these involve circumventing the BBB, whereas other are directed at osmotic opening of the BBB (35). Disruption of BBB allows uncontrolled passage of the drug into the brain surrounding the tumor rather than into the tumor itself, which may produce neurotoxic effects. Controlled passage through the BBB with targeted delivery to the tumor, as described in the section on nanobiotechnology-based delivery is safer and more effective.

#### Intra-arterial chemotherapy

Intraarterial delivery of chemotherapy to the brain provides a many-fold delivery peak drug concentration in the tumor as compared to the same drug dose given systemically because of damage to the blood-brain barrier and neovasculature in the tumor. However, randomized trials on patients with glioblastoma have not shown a survival advantage with intraarterial BCNU as compared to intravenous administration. The limitations of this technique are significant vascular and neurologic toxicity that can lead to visual loss, stroke, and leukoencephalopathy. Although toxicity of intra-arterial chemotherapy could be reduced by using carboplatin- and methotrexate-based regimens, further clinical studies are needed to determine its utility in the treatment of glioblastoma.

An animal experimental study has demonstrated the feasibility of direct delivery to brain and glioma tissue of cationic liposome after intraarterial injection via an intracarotid route during transient cerebral hypoperfusion (36). This may represent an effective method for delivering antiglioma agents to glioblastoma in humans with a drawback, e.g., cationic liposomes accumulate at higher concentrations in the peritumoral brain than in the tumor core and are retained for a longer time.

## Immune therapy

Immune therapy of cancer is progressing rapidly and has been applied to glioblastoma as well. Two types of immune therapies, monoclonal antibodies and vaccines, will be considered in this section. Immune gene therapy will be discussed under the section on gene therapy.

### Monoclonal antibodies

Monoclonal antibodies (MAbs) are used for glioblastoma treatment because of their high specificity and affinity for biological targets to improve immunotherapy and for antiangiogenic action by targeting growth factor receptors such as VEGFR, EGFR, and PDGFR (37). MAbs overlap with vaccines, another strategy for immunotherapy of cancer. Bevacizumab is the only approved MAb used for the treatment of glioblastoma. Several MAbs are under investigation.

#### Bevacizumab

Bevacizumab is a MAb that binds to VEGF and inhibits the growth of tumor blood vessels. It is approved for the treatment of several cancers including glioblastoma for a decade. A systemic review of literature shows that use of bevacizumab prolongs OS either alone or in combination with a cytotoxic agent by about 4 months in recurrent glioblastoma but not in primary glioblastoma (38).

#### MAbs targeting EGFR

The best-known example of an anti-EGFR MAb is cetuximab, which is approved for the treatment of other cancers but not for recurrent glioblastoma as phase II clinical trials failed to show its efficacy. MAbs with TK inhibitors targeting EGFR, a tyrosine kinase (TK), which is a receptor for therapeutic agents for glioblastoma such as T cells, oncolytic viruses, and nanoparticles is being investigated (39). Another, anti-EGFR MAb, nimotuzumab, was developed up to phase III clinical trials. It showed some efficacy in combination with other methods of treatment but was not developed further. There is a need for the development of a suitable MAb for targeting EGFR.

#### Delivery of MAbs for treatment of glioblastoma

Due to their large size, MAbs do not cross the BBB easily and nanobiotechnology-based strategies are required. These are described under the section on nanobiotechnology-based drug delivery.

### Immune checkpoint blockade

Immune checkpoint blockage is achieved by use of T cell inhibitory molecules such as anti-programmed cell death 1 (PD-1) antibody, which was first approved for the treatment of malignant melanoma. In contrast to use of cytotoxic T lymphocytes (CTL) against cancers, immune checkpoint blockade terminates immune response in a way that may activate exhausted CTL to destroy cancer (40). This approach is being investigated for several cancers including glioblastoma, but concern for adverse effects such as autoimmune diseases and prohibitive cost are drawbacks for wider applications.

Rationale for clinical trials of immune checkpoint blockade for newly diagnosed and recurrent glioblastoma is provided by results of preclinical studies (41). Furthermore, the concept of combination therapy involving with vaccine and immune checkpoint inhibitors is a promising strategy for treatment of patients with glioblastoma (42). An open-label pilot study will assess the safety, feasibility, and immunogenicity of a personalized neoantigen-based vaccine for enhancing CTL response again tumor cells plus adjuvant poly-ICLC combined with immune checkpoint inhibitors in patients with newly diagnosed, unmethylated glioblastoma (ClinicalTrials.gov Identifier: NCT03422094).

### Vaccines for glioblastoma

The earlier vaccines for glioblastoma were crude preparation made from patients own tumor tissue and results were disappointing. As of August 2018, 88 clinical trials of vaccines for glioblastoma are listed on the US Government web site for clinical trials^1^. Numerous trials of vaccines employing various strategies against glioblastoma are being conducted from phase I to phase III. Although some have shown promising results, none has come close to curing it. New biotechnologies have enabled better and more effective vaccines for glioblastoma. Some of the more promising of these will be discussed here.

#### DCVax

DCVax, a dendritic cell (DC)-based personalized cancer vaccine cancer type with purified tumor-specific antigens or tumor cell extracts derived from tumor at the time of resection. DCVax-Brain is approved in Switzerland for the treatment of glioblastoma. In the US, in a phase I trial of autologous DC vaccine, expression level of tumor-associated antigens on glioblastomas cells or glioblastoma stem cell population correlated with prolonged overall survival and progression-free survival (43). The ongoing open label clinical trial #NCT02146066, using already manufactured autologous tumor lysate-pulsed DC vaccine (DCVax-L, is studying patients who were not eligible for enrollment under protocol 020221 due to evidence of disease progression or post chemo-radiation pseudo-progression or lack of availability of adequate vaccine doses.

Vaccination with autologous tumor lysate-pulsed DCs in conjunction with toll-like receptor agonists administered as adjuvant therapy was tolerated well both in newly diagnosed and recurrent glioblastoma patients. Interestingly, mesenchymal gene expression profile, which is mostly defined by inflammation-associated gene signature, may identifies a subgroup of glioblastoma patients likely to respond to immune-based therapy (44).

Pre-conditioning with a potent recall antigen has been a viable strategy to enhance anti-tumor immune response. Experimental studies have shown that pre-conditioning the vaccine site with a potent recall antigen such as tetanus/diphtheria toxoid improves the efficacy of tumor-antigen-specific DCs (45). In a randomized trial on patients with glioblastoma, pre-conditioning with DCs or tetanus/diphtheria toxoid prior to vaccination with DCs pulsed with tumor specific cytomegalovirus phosphoprotein 65 RNA, a tumor-specific target had enhanced DC migration bilaterally and significantly improved survival (45).

One of the ongoing phase II clinical trials, # NCT02808364, is evaluating safety and efficacy of “Personalized Cellular Vaccine for Recurrent Glioblastoma” (PerCellVac2) in recurrent glioblastoma. This trial specifically uses immunization with autologous tumor cells, antigen pulsed DCsor allogeneic peripheral blood mononuclear cells. Results of this trial will be useful for determining the future course of action for cell-based immunotherapy in glioblastoma.

A randomized phase III trial NCT00045968 is evaluating long-term effects of addition of an autologous DCVax®-L vaccine to standard therapy for newly diagnosed glioblastoma, i.e., after surgery and chemoradiotherapy, patients receive temozolomide plus DCVax-L or temozolomide alone. Because of the cross-over trial design, nearly 90% of the patients have received DCVax-L so far and the vaccine has improved the survival rates of some patients and those with median overall survival of 40.5 months are being analyzed further (46). The trial is ongoing to enable continued study of glioblastoma patients who are living beyond the expected length of survival.

#### Epidermal growth factor receptor variant III as a vaccine target for glioblastoma

The epidermal growth factor receptor variant III (EGFRvIII) is an important vaccine target because its expression is tumor specific and has a promising role in immunotherapy of glioblastoma. Phase II clinical trials in patients with newly diagnosed glioblastoma showed that EGFRvIII-specific vaccine therapy improves progression free and overall survival (47). In a separate phase II study, rindopepimut (CDX-110, EGFRvIII peptide sequence conjugated to keyhole limpet hemocyanin) combined with standard adjuvant temozolomide chemotherapy improved progression-free and overall survival of glioblastoma patients (48). A pivotal, double-blind, randomized, phase II trial # NCT01498328 on bevacizumab-resistant patients with recurrent glioblastoma combining bevacizumab with rindopepimut was completed in 2017 but results have not been published.

#### Heat shock protein-based vaccine

Heat-shock proteins (HSPs) has been used to deliver a variety of tumor antigens to antigen presenting cells (APC) for immune stimulation. HSPPC-96 vaccine, based on internalization of HSP-96 by binding of to the CD91 receptor on DCs resulting in cleaved tumor peptides on major histocompatibility complex classes I and II, has been studied in patients with recurrent glioblastoma in a phase II, multicenter, clinical trial (49). A personalized polyvalent vaccine is obtained by purifying HSP-96 protein complexes from a patient's own tumor and administered for treatment. Results showed that the vaccine was safe but lymphopenia prior to treatment was a concern as it may reduce efficacy. An ongoing 3-arm randomized phase II clinical trial #NCT01814813 is comparing HSPPC-96 vaccine to vaccine in combination with bevacizumab and bevacizumab alone following surgical resection of glioblastoma.

#### Recombinant poliovirus

Recombinant nonpathogenic polio-rhinovirus chimera (PVSRIPO) targets the neurotropic poliovirus receptor CD155, which is abundantly expressed on glioblastoma cells, and penetrates these cells to cause lysis and release of tumor antigens as well as molecules recognized by cells of the natural immune response (50). This results in an influx of macrophages, monocytes, and DCs into the tumor to scavenge cellular debris, and released molecules present a pattern for natural killer (NK) cells and tumor antigens to induce effector T cells to kill more cells. This is an example of overlap of viral immune therapy and viral oncolysis of malignant tumors.

A phase II clinical trial confirmed the absence of neurovirulent potential following intratumoral infusion of PVSRIPO in patients with recurrent glioblastoma and observed that survival rate of treated patients was higher at 24 and 36 months than the rate among historical controls (51). Patients in the trial received polio vaccine before treatment to ensure poliovirus immunity at the time of instillation of PVSRIPO into the tumor and reduce the risk of spread of the virus beyond the tumor. A question that has been raised is if viral immunity may reduce the efficacy of the vaccine. There is a potential risk of restoration of replication competence of virus *in vivo*.

## Nanobiotechnology-based innovations for targeted delivery of therapy for glioblastoma

Nanobiotechnology-based strategies for drug delivery to cancer are described in detail elsewhere (52). Some of these can be applied to glioblastoma and examples are given here. Nanoformulations of anticancer drugs enable targeted and efficient delivery to tumors through the blood-brain barrier (BBB) with lesser dosage of anticancer drugs than conventional formulations and reduce toxicity of chemotherapy.

### Micelles for delivery of chemotherapy to brain tumors

Micelles can be used as carriers of anticancer drugs such as temozolomide (TMZ) to enhance delivery to glioblastoma. In an experimental study on a mouse model of implanted glioblastoma, pH-responsive micelles containing distearoyl phosphoethanolamine-PEG-2000-amine, N-palmitoyl homocysteine, and platelet-derived growth factor (PDGF) peptide as well as Dylight 680 fluorophore on the surface for targeting were used for delivery of TMZ (53). This resulted in specific uptake and accumulation of TMZ in tumors with increased destruction of tumor cells compared to delivery with untargeted micelles. Thus, micelle-based drug carrier systems have a potential for targeted delivery of anticancer drugs to glioblastoma to reduce their systemic toxicity.

### Nanoparticles for delivery of drugs to glioblastoma across BBB

Nanoparticles are promising tool for targeted delivery of oncology drugs. Nanoparticles made of poly(butyl cyanoacrylate) (PBCA) or PLGA coated with polysorbate 80 or poloxamer 188 have been shown to transport antitumor drug doxorubicin across the BBB (54). In an orthotopic model of glioblastoma in rats, these particles loaded with doxorubicin, significantly improved survival with complete tumor remission observed in 20–40% animals (54). This nanoparticle approach of drug delivery reduced dose-limiting cardiotoxicity and the testicular toxicity of dauxorubicin. The mechanism of nanoparticle aided delivery of dauxorubucin across the BBB remains unclear. However, it is likely that certain plasma apolipoproteins adsorbed by nanoparticles may interact with receptors on brain capillary endothelium to suppress drug exclusion activity (55).

Iron oxide nanoparticles are particles with superparamagnetic properties, also known as superparamagnetic iron oxide nanoparticles (SPION) have been useful in MRI to locate brain tumors with precision and to target chemotherapeutic drugs into the tumors. Folic acid (FA) has been used as the targeting agent combined with polyethylene glycol (PEG) serving to improve biocompatibility and cellular uptake of nanoparticles. These nanoparticles has applicability beyond MRI mediated tumor detection because a variety of small molecules to target receptor tyrosine kinases on tumor or chemotherapy drugs, can be attached to these nanoparticles to facilitate delivery and efficacy.

A biodegradable and nontoxic biopolymer is a universal delivery nanoplatform for design of nanomedicines for intravenous treatment of for malignant brain tumors. A polymeric conjugate of a MAb targeting the brain-tumor barrier for crossing it and attached to an antisense oligonucleotide that inhibits tumor angiogenesis by specifically blocking the synthesis of a tumor neovascular trimer protein, laminin-411, is released into the target tumor cell cytoplasm via pH-activated trileucine (56). This is a promising strategy for treatment of glioblastoma that should be tested in clinical trials.

### Nanoparticle aided delivery of chemotherapy

**Figure 1:**
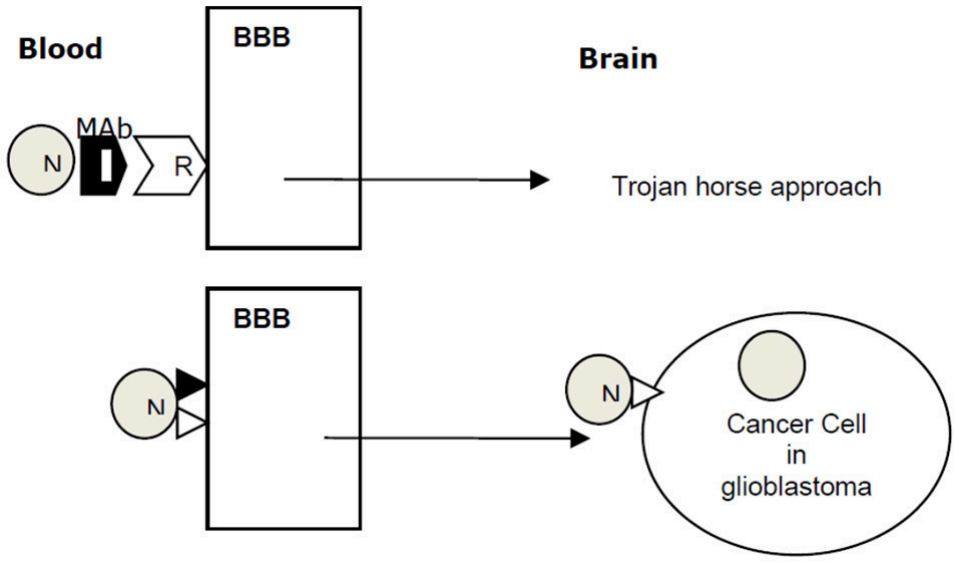
A concept of targeted drug delivery to glioblastoma across the BBB. Nanoparticle (N) combined with a monoclonal antibody (MAb) for receptor (R) crosses the blood brain barrier (BBB) into brain by Trojan horse approach. N with a ligand targeting BBB traverses the BBB by receptor-mediated transcytosis. Ligand ▸ docks on a cancer cell receptor N⊳ and delivers anticancer payload to the cancer cell in glioblastoma.

A concept of targeted drug delivery to glioblastoma across the BBB is shown in Figure 1. Several techniques have been shown to improve drug transport across BBB, many of these techniques are designed to disrupt BBB, which compromises the ability of brain microvasculature to prevent entry of harmful toxins in to the brain. Nanoparticle-based delivery of anticancer drugs across the BBB is a promising approach. Polymer and lipid nanoparticles are frequently used as nanocarriers for anticancer drugs. A phase I clinical trial of nanoliposomal irinotecan in recurrent glioblastoma has been completed (NCT00734682).

### Controlled delivery of BCNU by poly-lactic acid nanoparticles

Biodegradable poly-lactic acid (PLA) nanoparticles conjugated with transferrin, an iron-transporting serum glycoprotein were loaded with BCNU for targeted delivery to transferrin receptor expressing glioma cells (57). Efficacy evaluation in C6 tumor-bearing rats, BCNU-loaded PLA nanoparticles showed superior cytotoxicity, and led to prolonged survival animals of as compared to conventional BCNU therapy (57).

### Targeted minicells for nanoscale delivery of chemotherapy to glioblastoma

Minicells are achromosomal cells formed in certain mutants of rod-shaped bacteria, are products of aberrant cell division, and contain RNA and protein, but little or no chromosomal DNA (58). Designing minicells loaded with anti-cancer drugs and coated with antibodies to target tumor cell specific receptors is relatively new technique. Targeted minicells measuring 400 nm enter cancer cells via receptor-mediated endocytosis and can be loaded with therapeutically significant concentrations of chemotherapeutics. As proof of principle, clinical applicability of minicells was shown in dogs with late stage spontaneous brain cancer, where targeted minicells loaded with doxorubicin were safely administered achieving clinical activity in terms of tumor regression (58). This study demonstrated promising results and potential clinical application of minicells for drug delivery for treatment of patients with glioblastoma. Based on this, a phase I clinical evaluation of EGFR-targeted, doxorubicin-loaded minicells (EGFR(V)-EDV-Dox) was performed in human patients with recurrent glioblastoma in Australia and another phase I trial # NCT02766699 is currently under progress in the USA.

### Targeting MAbs attached to the surface of the nanocarrier

The surface of nanocarriers is functionalized with targeting MAbs as ligands to promote drug delivery to tumors with corresponding receptors. One problem with this approach is that when nanocarrier is exposed to biological fluids such as plasma, its surface is covered with various biomolecules forming a protein corona, which masks the targeting ability of the nanoparticle. A pre-adsorption process has been used to attach targeting MAbs to the surface of the nanocarrier, a capsule containing the anticancer drug, which prevents the formation of biomolecular corona and pre-adsorbed MAbs remain functional (59). The authors concluded that this is an efficient method for attaching targeting MAbs to the surface of nanocarriers.

## Innovations in radiotherapy

Radiotherapy is usually combined with chemotherapy following surgery in different sequential combinations. There is no convincing evidence that addition of radiotherapy to chemotherapy increases survival in glioblastoma. Traditionally radiation therapy following surgery was whole cranium radiation, which exposed the normal brain to radiation with adverse effects such as cognitive impairment. Current practice is to use “focal” or “limited-field” radiation therapy covering 2–3 cm around the tumor, interstitial brachytherapy, and fractionated radiotherapy.

### Fractionated radiotherapy

Fractionated radiotherapy means splitting the total radiation dose into several fractions that are given over several weeks in combination with chemotherapy. The rationale for using a fraction of the total radiation dose enables normal cells surrounding the tumor to recover between treatments. Adverse effects of full dose radiation in a single session are reduced by fractionated therapy over several weeks. In case of glioblastoma, 30 fractions of radiotherapy dose of 60 Gy with adjuvant temozolomide is the current standard of treatment (60). This approach is included in the American Society for Radiation Oncology Guideline, which is endorsed by the American Society of Clinical Oncology (61). Fractionated radiotherapy can be combined with immunotherapy for glioblastoma. Preclinical evidence indicates that hypofractionated radiotherapy of glioblastoma can prime the immune system to enhance the effect of immune therapy (62).

### Brachytherapy

Interstitial brachytherapy involves placement of radioactive isotopes such as ^125^I seeds in the tumor cavity after resection to deliver high dose radiotherapy to the residual tumor with minimal radiation exposure in the surrounding brain tissue as compared to external-beam radiation. Nevertheless, radiation may leak into the surrounding healthy brain and may accumulate in other organs via systemic circulation. Three-dimensional iridium-192 (^192^Ir) high-dose-rate brachytherapy (Ir Knife) allows intratarget dose escalation with superior conformity, which compares favorably with linear accelerator-based stereotactic radiosurgery with a steeper dose fall off at margin of the tumor (63). In non-operable glioblastomas, low-dose ^125^I brachytherapy may be administered by a stereotactic device. Stereotactic ^125^I brachytherapy in combination with temozolomide chemotherapy was shown to be effective for treatment of thalamic glioblastoma (64).

Innovations in brachytherapy under investigation include other isotopes with prolonged delivery of higher doses of radiation, and combination of radioactive isotopes with MAbs as ligands for receptors expressed on the surface of tumor cells for selective delivery of radiation therapy. Radiolabeling can further enhance the assessment of effects of therapy by brain imaging. For example, ^32^p (phosphorus-32)-chromic phosphate-polylactide-co-glycolide (32P-CP-PLGA) for controlled release of the isotope, combined with radiotracer ^68^Ga-3PRGD_2_, which targets integrin αvβ3 receptors or the tumor as well as neovasculature, has shown promising results of brachytherapy in a rat model of transplanted glioblastoma (65).

### Heavy particle radiation

Proton beam radiation therapy due to its “Bragg peak effect,” reduces exposure to the surrounding brain, i.e. steeper dose “drop-off” relative to photon radiation therapy. In principle, proton beam therapy can be considered safer than traditional photon radiation therapy; however, it is uncertain if proton beam radiation has any better antitumor effects than photon radiation therapy.

Gamma Knife radiosurgery (GKRS) is a type of non-invasive stereotactic radiosurgery for delivery of a high dose of radiation to a tumor. GKRS is suitable for recurrent glioblastoma as it spares the poorly demarcated healthy brain tissue surrounding the tumor from radiation-induced necrosis (66). Drawbacks of GKRS are that it does not reach areas of the tumor that are not well detected by MRI, and radiation-induced edema has been reported in nearly 30% of patients who received high radiation doses (67). Concomitant administration of bevacizumab was shown to prolong patient survival, while also significantly reduced adverse effects of radiation (67).

### Boron neutron capture therapy

In boron neutron capture therapy (BNCT), another form of heavy particle radiation, irradiation of the glioblastoma with low-energy neutrons is followed by the boron emits an alpha-particle that deposits high energy over a short distance systemic administration of a nonradioactive boron isotope. Monte Carlo modeling for BNCT of glioblastoma has shown that it increases the efficacy in destruction of tumor, but extension of the CTV margin may not increase the outcome of treatment significantly (68).

### Enhancing effect of radiotherapy by hyperbaric oxygen

Hyperbaric oxygen (HBO) is known to enhance the effect of radiotherapy by counteracting hypoxia within tumors, a fundamental feature of cancer cells that limits efficacy of radiation therapy (69). HBO is also used for treatment of radiation induced necrosis of normal tissues around tumor treated with high-dose radiation therapy. In a study on glioblastoma cells that were exposed to HBO at 1.3 ATA (atmospheric pressure absolute) showed that HBO treatment reverses radiation-induced enhanced mobility of tumor cells (70). In the first report of a clinical pilot study, results of radiotherapy combined with HBO in glioblastoma were compared with those of radiotherapy without HBO and all patients receiving HBO with radiotherapy showed more than 50% regression of the tumor (71). Several studies have reported that radiotherapy immediately after HBO therapy is safe and effective in patients with glioblastoma and may protect normal brain tissues from radiation injury (72).

## Local destruction of tumor

### Oncolysis by genetically modified viruses

Viral oncolysis is a targeted therapy and oncolytic viruses are genetically modified to specifically lyse tumor cells. They are replication-selective rather than replication-defective viral vectors. Oncolytic viruses differ from viral vectors in that they increase in number in tumor cells and lyse the cells directly, not by transducing specific genes. Studies both in a mouse glioma model as well as on glioma stem-like cells from patients suggest that the efficacy of viral oncolysis HSV type 1 may be enhanced when used in combination with inhibitors of histone deacetylases or other proteins that modulate cellular trafficking of these therapeutic viruses (73). Most of the oncolytic viruses currently in clinical trials are derivatives of adenovirus or herpes simplex virus (HSV) type I. T-Vec (talimogene laherparepvec), an oncolytic HSV-1 armed with GM-CSF, has been approved as the first oncolytic virus therapy in the USA and Europe. A phase II clinical trial of G47Δ, a third-generation oncolytic HSV-1 designated as breakthrough therapy, is ongoing in glioblastoma patients in Japan (74).

Oncolytic virotherapy is administered into the residual tumor after resection of glioblastoma and may not penetrate all tumor cells. Some of the precautions that need to be observed during clinical introduction of oncolytic therapy include the following (75):

- Oncolytic virus delivery should be completely optimal and safe.
- Viruses should be monitored for replication or dissemination beyond the tumor.
- Reaction of immune system of the patient to oncolytic viruses should be closely monitored.

### Genetically modified bacteria for tumor-specific lysis

Genetically modified bacteria can be used for selective destruction of glioblastoma. Genetically altered strains of bacteria such as Salmonella have been used as bacterial vectors for preferential delivery of anticancer drugs to solid tumors. Historically, bacteria have been used as oncolytic agents for malignant brain tumors. The most promising approach to glioblastoma was the use of genetically engineered bacteria that selectively destroys tumor cells while sparing the normal brain tissue (76). Genetically altered bacteria can be maintained in the confines of the brain tumor, where they thrive and unable to survive if they escape into brain or other tissues. Once the tumor destruction is completely, a bactericidal drug can eliminate bacteria. Refinements in gene editing can bring this concept closer to application for treatment of glioblastoma in patients.

### Hyperthermia

Hyperthermia, i.e., raising the tumor tissue temperature to 41–46°C damages tumor cells by changes such as protein misfolding and disruption of signaling pathways leading to apoptosis. Hyperthermia can be induced by microwaves, infrared irradiation, ultrasound, and magnetic nanoparticles. In addition to destroying tumor cells, hyperthermia can also be used for targeted delivery of drugs to glioblastoma by thermosensitive release from drug carriers guided to enter the tumor. Gold nanoparticles can be used both as contrast agent for MRI and for photothermal therapy, e.g., gold nanorods have been used for thermal ablation of glioblastoma (77).

### Tumor treating fields

Tumor treating fields (TTF), a non-invasive wearable technology, is based on low-intensity alternating electric fields, which disrupt mitosis and inhibit tumor growth. TTF is approved for glioblastoma by the FDA in US. A systematic review of preclinical studies and clinical trials of TTF for glioblastoma has shown that TTF was as effective as chemotherapy but the combination of both prolonged overall survival and progression-free survival of glioblastoma without systemic adverse effects (78). Combinations of TTF with radiation therapy, targeted chemotherapy as well as immunotherapy remain to be explored.

## Gene therapy

No gene therapy for glioblastoma has been approved in the US or Europe yet, but 77 clinical trials are listed on US Government web site^1^ as of August 2018. Gene therapy may be used in conjunction with surgery or as an alternative to chemotherapy/radiotherapy or in cases of recurrences following excision as well as chemotherapy-resistant tumors. With improvement in diagnostic imaging and methods of delivery, it feasible that small tumors detected very early may be treated noninvasively or by minimally invasive gene therapy procedures.

The first clinical trial of the herpes simplex virus, thymidine kinase, and ganciclovir gene therapy for glioblastoma was conducted a quarter of century ago (79). However, by the end of twentieth century, this gene therapy approach was discontinued from further development after completion of phase III clinical trials as it failed to show efficacy in patients (80). The results in patients were less striking than in experimental animals. Initially there was reduction in the size of the tumor, but the tumors resumed the growth and the OS was not extended. Various innovations in gene therapy have been employed since then. The number of current preclinical research projects worldwide to find a cure for glioblastoma exceeds 100.

A classification of gene therapy approaches to glioblastoma is shown in Table 3. Discussion in the article does not follow or include all the technologies or follow the same order due to overlap with other approaches. Some technologies relevant to the topic of this article will be described here.

**Table 3:** Strategies for gene therapy of glioblastoma.

**Viral vector mediated insertion of drug sensitivity genes**
Direct intratumoral injection of genetically modified neurotrophic viruses
Insertion of drug sensitivity genes
Suicide gene therapy: HSV thymidine kinase
Use of *E. coli* gpt gene to sensitize glioma cells to prodrug 6-thioxanthine
**Use of viral vectors containing radiation inducible promoters**
**Regulated toxin gene therapy**
Baculovirus as diphtheria toxin gene vector
**Transfer of apoptosis inducible FADD/MORTI gene**
**Gene transfer into brain tumors by using targeted adenoviral (Ad) vectors**
Single chain antibody combined with Ad vector and targeted to EGFR receptor
**Gene transfer into brain surrounding tumors by using targeted adeno-associated viral (AAV) vectors**
Transduction of normal cells in the brain with an AAV vector encoding interferon-β (IFN-β)
**Selective oncolysis by genetically engineered microorganisms: bacteria and viruses**
**Immunogene therapy**
**Cell-based gene therapy**
Mesenchymal stem cells engineered to produce therapeutic molecules
Neural stem cells engineered to produce therapeutic molecules
Grafting of stem cells producing therapeutic molecules such as IL-4 gene
**Growth factor manipulation**
Apoptosis induced by introduction of gene for nerve growth factor receptor TrkA
Inhibition of epidermal growth factor receptor associated tyrosine kinase receptor
**Antiangiogenesis approaches directed against tumor blood vessels**
**Oncogene antagonism: anti MYC oncogene MAD therapy**
**Insertion of tumor suppressor genes**
Transfer of wild type p53 or p27
Retinoblastoma gene transfer
**Antisense therapy**
Blocking of action of transforming growth factor β2 by triplex forming oligonucleotides
Episome-based antisense cDNA transcription of insulin like growth factor 1
Antisense vascular endothelial growth factor.
Oligodenucleotides targeted to tumor necrosis factor
**RNA interference (RNAi)-based approaches**
siRNA directed against EGFR and its variants
siRNA directed against PI3K/Akt signaling pathways
Telomerase inhibition by RNAi

### Choice of a viral vector for gene therapy of glioblastomas

Three types of viral vectors have been found to be efficient for delivery of gene therapeutics in most of the clinical trials: (1) retrovirus, (2) herpes virus, and (3) adenovirus. An adenoviral vector has the following advantages for treating glioblastoma:

- It can be easily produced at high titers.
- It can be directly injected as purified particles into brain tumors without the need for virus producing cells.
- It does not integrate into the host genome. This avoids the risk of insertional mutagenesis.
- Gene delivery by an adenovirus is independent of the host cell cycle. This feature allows higher transduction rates than with retroviruses because only a small number of glioma cells are replicating at any given time.
  Disadvantages of adenoviral vectors are as follows:
- Actual release *in vivo* cannot be quantified.
- Antigenicity of viral proteins; this might limit repeated treatments. This problem has been seen in gene therapy trials for cystic fibrosis, but it is uncertain if this problem would occur in the brain, as it is an immunologically privileged site.
- Unlike retrovirus vectors, they infect all cells of the brain. This lack of discrimination could result in significant toxicity to the normal surrounding brain. This can be remedied by use of tissue-specific promoters. One example is glial fibrillary acidic protein, a gene that is found in glial cells. Transfer of a vector containing a cytotoxicity gene under the control of a glial fibrillary acidic protein promoter should result in expression only within the cells that normally synthesize glial fibrillary acidic protein.

Glioblastoma expresses high levels of type 2 somatostatin receptors, which can be targeted for improving transduction efficiency in these tumors. An adenoviral vector was designed based on the introduction of the full-length somatostatin somatotropin release-inhibiting factor sequence into it, and it was shown that low doses of this vector were sufficient for infecting high-grade human glioblastoma cells with marked enhancement of gene expression (81).

Results of an open-label, randomized, phase III trial of locally applied adenovirus-mediated gene therapy with herpes-simplex-virus thymidine kinase (sitimagene ceradenovec) followed by intravenous ganciclovir in patients with newly diagnosed glioblastoma after resection can increase time to death or reintervention, but not overall survival (82). Further clinical trials are in progress, and it will be the large randomized phase III controlled clinical trials that will provide evaluation of the success of gene therapy for the treatment of glioblastoma (83).

### Antiangiogenic gene therapy

Gene therapy strategies, developed to disrupt normal function of vascular endothelial growth factor receptors, have been successful in experimental models to suppress tumor angiogenesis and growth (84). Some of the antiangiogenic gene therapies are described here briefly.

#### Targeting endothelial vasculature in brain tumors

VB-111 (ofranergene obadenovec), a genetically modified adenovirus, selectively targets endothelial cells in neovasculature of tumors (85). VB-111 inhibits vascular density in mouse models bearing glioma xenografts, which justifies its clinical development as a treatment for glioblastoma (86). A phase III trial #NCT02511405 for recurrent glioblastoma is studying VB-111in combination with bevacizumab as well as without it.

#### Combined antiangiogenesis approach

Systemic adenoviral delivery and sustained production of endostatin and soluble vascular endothelial growth factor receptor-2 can slow glial tumor growth in animal models by both reducing cell proliferation and increasing tumor apoptosis (87).

### Gene therapy for reducing adverse effects of chemotherapy

Raising the dose of chemotherapy to improve clinical efficacy is limited by toxicity, particularly myelosuppression, and neurotoxicity. Gene therapy using mutant MGMT (P140K) gene-modified hematopoietic stem cells can reduce the toxic effects of chemotherapy on hematopoietic cells, and autologous P140K-modified hematopoietic stem cells transplantation has been used in glioblastoma patients with poor prognosis prior to administration of multiple cycles of chemotherapy, resulting in increase of survival without adverse effects (88).

Glioblastoma frequently develops resistance to temozolomide, which can be overcome by adding O6-benzylguanine (O6BG), but the combination produces myelosuppression. Results of a prospective clinical trial have shown that gene therapy P140K-modified hematopoietic stem cells to confer O6BG resistance improves chemotherapy tolerance and outcome in these patients (89).

### Nanobiotechnology for improving delivery of gene therapy

Nanobiotechnology, particularly use of nanoparticles, has improved drug delivery in cancer, and this technology can be applied to gene therapy of glioblastoma (90). A nanoparticle preparation using low molecular weight polyethylenimine, modified with myristic acid, and complexed with DNA, has been used successfully for targeted delivery of gene therapy for glioblastoma (91).

## RNA interference therapy of glioblastoma

Nearly 40–50% of glioblastoma tumors show alterations (amplification, truncation, or mutations) of epithelial growth factor receptor (EGFR) resulting in an uncontrolled multiplication and expression of gene encoding normal EGFR or truncated form called EGFRvIII. Because of the delivery problems with commonly used pharmacologic inhibitors of EGFR, RNA interference (RNAi) would be an ideal approach to target EGFRvIII to destroy brain cancer cells and spare healthy cells. Similarly, small-interfering RNA (siRNA)-based downregulation of DNA repair protein MGMT in tumor cells can enhance the chemosensitivity of malignant gliomas against temozolomide (92). The siRNA applied to glioblastoma cells *in vitro* was shown to reduce gene expression of EGFR and β-catenin and significantly inhibit their migratory as well as invasive ability (93). This is potentially an effective therapy for human glioblastoma and warrants further study *in vivo*. Knockdown of DNA repair protein apurinic endonuclease 1 by nanoparticle-based delivery of a siRNA has been demonstrated to increase sensitivity to radiotherapy in a genetic mouse model of glioblastoma resulting in prolonged survival (94).

## Cell therapy of glioblastoma

### Stem cell therapy for glioblastoma

Implanted neural stem cells (NSCs) are known to migrate to glioblastomas and distribute inside the tumor in experimental animals, indicating their potential use as delivery vehicles for targeted therapeutics including gene therapy. The mechanism of the attraction of NSCs to the tumor has not been elucidated. Various proposed mechanisms that drive NSC migration include multiple factors such as chemoattractant molecules released by the tumor cells and inflammatory or degenerative changes in tumor microenvironment (95).

Intratumoral injection of interleukin (IL)-12 secreting NSCs in mice bearing intracranial gliomas significantly prolongs survival and leads to long-term antitumor immunity. Tumoricidal potency of IL-12 with the extensive tumor tracking capability of NSCs result was thought to render exceptional therapeutic benefit. Genetically engineered NSCs have been shown to specifically target glioblastoma cells after traveling through brain parenchyma and hinder tumor growth through local activation of cyclophosphamide-activating enzyme cytochrome p450 2B6 (96). Significance of the NSC-based gene therapy for brain tumor is that it exploits the tumor-tropism of these cells to mediate effective, tumor-selective therapy for brain cancer (97).

Human embryonic stem cell (hESC)-derived engineered mesenchymal stem cells (MSCs) have been shown to inhibit tumor growth and prolong survival after they were injected directly into the glioblastoma xenografts in the brains of mice who received concomitant prodrug ganciclovir (98). A preclinical study using MSCs engineered to express cytosine deaminase has shown that that stem cell-based gene therapy may be effective against glioblastoma stem cells (99). The authors proposed starting clinical studies in human patients based on encouraging results of preclinical studies of stem cell-based gene therapy for glioblastoma.

Cancer stem cells (CSCs), which are a type of tumor cells with self-renewal ability and high tumorigenicity, which contribute to the high rates of recurrence after treatment as well as development of resistance to treatment in glioblastoma patients. Therapeutic strategies for CSCs include inhibition of CSC-specific pathways and receptors by agents that increase sensitivity of CSCs to chemotherapy and radiotherapy, virotherapy, and gene therapy (100). A subset of CSCs in glioblastoma is marked by cell surface expression of CD133, a glycosylated pentaspan transmembrane protein. CD133-LV (lentiviral) is a novel tool for the selective genetic manipulation of CSCs in glioblastoma that can be used to precisely study the role of CSCs in tumor biology and therapy resistance (101).

Aptamers that specifically bind to tumor-initiating cells were identified by adopted Cell-Systematic Evolution of Ligands by Exponential Enrichment (Cell-SELEX) technique (102). These aptamers selectively bind on and internalize into cells that self-renew, proliferate, and initiate tumors. Because they can be modified to deliver payloads, aptamers could selectively target or facilitate imaging of tumor-initiating cells to improve therapeutic outcomes in individual patients.

### CAR-T cell therapy of glioblastoma

Chimeric antigen receptors (CAR)-T cells combine the antigen binding site of a MAb with the signal activating machinery of a T cell, freeing antigen recognition from MHC restriction and thus breaking one of the barriers to more widespread application of cell therapy. CAR-T technology uses retroviral or lentiviral vectors to engineer CARs which graft an arbitrary specificity onto an immune effector cell such as a T cell. These modified T cells are then transferred to the patient. Targeting with CAR-T cells is like that with MAbs with additional advantages of active passage to tumor sites, *in vivo* expansion as well as long duration of action, and possibility of gene transfer for counteracting tumor immune evasion (103).

Regression of glioblastoma has been reported following multiple infusions of CAR-T cells targeting the tumor-associated antigen IL-13 receptor alpha 2 (IL13Rα2) in a patient with recurrent multifocal glioblastoma and no toxic effects were observed into the resected tumor cavity followed by infusions into the ventricular system (104). Intravenous delivery of a single dose of autologous CAR-T cells targeting EGFRvIII mutation in patients with recurrent glioblastoma has been shown to be feasible and safe, without evidence of off-tumor toxicity or cytokine release syndrome (105).

### Cell therapy for chemobrain

Chemotherapy for glioblastoma can produce “chemobrain” with severe cognitive dysfunction that can persist after the cessation of treatment. Pathomechanism is chemotherapeutic agent-induced inflammation in the hippocampus, which is involved in learning and memory. This inflammation can destroy neurons and other cell types in the brain. A study in rodent model of chemobrain, cognitive impairments due to chronic cyclophosphamide treatment resolved after intrahippocampal transplantation of human NSCs, which triggered the secretion of neurotrophic growth factors for regeneration and reduction of inflammation (106). A clinical trial to analyze the safety of such approaches is feasible.

## Combination of innovative with conventional therapies

### Combination of gene therapy with chemotherapy

An example of combination of gene therapy and chemotherapy is clinical trial # NCT02414165 titled “Toca 511 & Toca FC vs. Standard of Care in Patients With Recurrent High Grade Glioma.” This is a randomized, open-label phase II/III trial of combination treatment using retroviral vector Vocimagene amiretrorepvec (Toca 511), a replicating virus that only infects actively dividing tumor cells to deliver the gene for enzyme, cytosine deaminase (CD), and sustained release 5-fluorocytosine (Toca FC), the prodrug of the chemotherapy 5-fluorouracil. Once inside tumor cells, CD converts the prodrug to 5-fluorouracil to, which destroys them as well as immunosuppressive myeloid cells, enhancing the patient's immune system to recognize and attack the cancer cells. As of August 2018, the trial is still ongoing but analysis of a subset of phase I (selected to proceed to phase III) showed that durable response rate of 21.7% in patients with recurrent glioblastoma who were treated with a gene therapy combination were alive 33.9+ to 52.2+ months after treatment (107).

### Combination of gene therapy and CAR-T cell therapy for glioblastoma

Regulatory T cells (Tregs), tumor associated macrophages (TAMs) and myeloid derived suppressor cells (MDCSs) are immunosuppressive cells in microenvironment of glioblastoma, and they inhibit cytotoxic T cells anticancer functions, thus reducing the efficacy of immunotherapy. Tumor cells in glioblastoma acquire new mutations after treatment and make it resistant to further treatment, which requires combination of gene therapy for glioblastoma with enhancement of the immune system's ability to fight it, e.g., immune checkpoint blockade combined with gene therapy prevents cancer cells from hijacking the host immune system (108). This study also supports the concept that heterogeneity of the glioblastoma should be taken into consideration of a personalized approach to therapy.

## Animal models for testing innovative therapies for glioblastoma

Translation of preclinical research into clinical application is limited by lack of a suitable animal model of glioblastoma. The most commonly used model in the past has been a subcutaneous xenograft of human glioblastoma cell line in an immunodeficient rodent model. A limiting factor in intracranial xenografts is the short survival. Malignant brain tumors induced by injection of chemicals and viruses are not suitable for glioblastoma studies. The most appropriate rodent model is transgenic as genetic engineering allows selective introduction of mutations relevant to human glioblastomas. Advances in genome-wide sequencing will enable creation of mouse models of glioblastoma to reproduce gene mutation patterns of human glioblastoma that are suitable for preclinical testing of personalized therapies (109).

Spontaneously occurring glioblastoma in the dog resembles glioblastoma of human and immune system of dogs is also somewhat like that of humans. CT-guided stereotactic techniques for drug delivery and tumor biopsy from tumors in the brain have been developed for use in dogs. Thus, dogs with spontaneous brain cancers offer a scientifically and ethically attractive system for preclinical evaluation of novel interventions for glioblastoma (110). Clinical trials of glioblastoma therapies in dogs have already been conducted and the findings are expected to benefit human patients (111, 112).

## Personalized/precision approaches

Personalized medicine, also referred to as precision medicine is a type of medical care in which treatment is customized for an individual patient taking into consideration the genetic and environmental factors that influence response to therapy. Advancements in genomic/proteomic technologies have been crucial in development of personalized medicine, but other technologies such as metabolomics and adoptive immunotherapy also have contributed significantly to this effort. Personalized medicine is perfectly integrating technological advancements such as nanomedicine for understanding pathobiology of glioblastoma and management of disease in clinic (113).

### Brain cancer chip for personalized drug screening

Selection of an anticancer therapeutic best suited for an individual's glioblastoma is challenging. Conventional 2D cell cultures used in current drug discovery do not reflect the tumor and its 3D environments. A novel 3D brain cancer chip composed of photo-polymerizable poly(ethylene) glycol diacrylate (PEGDA) hydrogel, uses cultured glioblastoma cells that form 3D cancer tissues are is promising technology for drug screening (114). PEGDA hydrogel is permeable to water and biomolecules, so it enables “smart release” of chemicals carried on the chip for studying drug response in the surrounding 3D environment. Realistic cell–cell/cell–matrix interactions are like *in vivo* environment for drug screening. This chip was used to test combined treatment with two FDA-approved anticancer drugs—pitavastatin and irinotecan. High-throughput drug screening and massive parallel testing of drug response was possible using only a tiny sample from the tumor biopsy to determine the drug combinations and their dosages likely to be effective for personalized therapy of glioblastoma.

### Countering drug resistance in glioblastoma

A major obstacle to effective treatment is *de novo* or acquired resistance to standard-care therapies as well as for targeted therapies, EGFR, or mTOR inhibitors. Despite its nearly universal activation of mammalian target of rapamycin (mTOR) signaling in glioblastoma, tumors are resistant to mTOR-targeted therapy (115). Molecular analysis of glioblastoma cell lines, patient-derived cell cultures and clinical samples from phase I clinical trials suggest that the expression of promyelocytic leukemia (PML) gene may be responsible for resistance to cytotoxicity of mTOR inhibition (115). Consistent with this hypothesis, blockade of mTOR signaling by inhibitors of mTOR or EGFR promote nuclear PML expression in glioblastoma cells. Furthermore, studies using genetic ablation or overexpression techniques also demonstrate that PML abates cytotoxic effects of mTOR or EGFR inhibition; while inhibitors of PML restored sensitivity to mTOR kinase inhibitor tested *in vivo*. These results indicated role for PML in mTOR and EGFR inhibitor resistance and provide a strong rationale for testing this combination in clinical trials.

Because intratumor heterogeneity in glioblastoma is an important factor that contributes toward treatment failure and drug resistance. Genomic analysis has been used to uncover intratumor heterogeneity in terms of copy number alterations in EGFR and CDKN2A/B/p14ARF as early events, and aberrations in PDGFRA and PTEN as later events during progression of cancer (116). Striking findings of this study revealed patient-specific patterns of cancer evolution that can be useful for designing more effective personalized therapy.

Enhanced DNA repair enables cells to survive the genotoxic effects of chemotherapy. Akt3, found in glioblastoma, enhances tumor progression by activating DNA repair pathways, leading to enhanced survival of human glioblastoma cells following radiation or temozolomide treatment (117). This finding has potential applications as blockade of Akt3 may help prevent or alleviate DNA repair-mediated therapeutic resistance.

### Genomic analysis as a guide to personalized therapy of glioblastoma

Gene expression profiling combined with mutation analysis plays an important role in the development of rational targeted therapies for glioblastoma. Application of these approaches is limited by spatial and temporal heterogeneity of glioblastoma. An analysis of data from patients with glioblastoma showed that samples from the same tumor are likely to have same genomic and expression signatures, whereas geographically separated, multifocal tumors or recurrent tumors often represent clonal variability (118). In this study, patient-derived glioma cells showed that therapeutic response correlated with genetic similarity while drug-response was heterogeneous in multifocal tumors enriched with PIK3CA mutations suggesting that an integrated genomic analysis of biopsies from multiple foci in the tumor can guide targeted therapeutic interventions for patients with glioblastoma.

### Induced neural stem cells for personalized therapy of glioblastoma

Induced neural stem cells (iNSCs) derived from a patient's skin cells are ideal for personalized cell therapy of glioblastoma. Genetically engineered iNSCs with tumoricidal gene products retain the capacity to differentiate while induced apoptotic response in co-cultured human glioblastoma cells. These iNSCs also demonstrate unique capability to reach distant tumor sites in the murine models of glioblastoma to deliver anticancer molecule TRAIL (TNF-α-related apoptosis-inducing ligand) with resulting increase in survival (119). The use of iNSCs could lead to highly selective delivery of therapeutics to brain tumors, reducing systemic toxicity and improving efficacy. iNSCs may effectively bypass the BBB and attack glioblastoma (120). Considerable preclinical work needs to be done before moving iNSCs into clinical trials.

## Concluding remarks

Introduction of new technologies has improved the outlook for cure of glioblastoma although much remains to be done. Prolongation of survival time does not necessarily translate into eventual prospects of cure. Reduction of size of tumor alone is not enough as recurrences and rapid progression of the tumor eventually kill the patient. Improvements in targeted delivery of therapies is important for efficient and safe destruction of the tumor. MAbs with ligands for receptors on tumor surface have been used for targeted delivery of therapies. Although several receptors have been identified on glioblastoma cells, they may occur in other organs. A future challenge is discovery of receptors that are unique to glioblastoma and a combination of receptors may be needed for selective delivery to the tumor.

Nanobiotechnology has provided important diagnostic and therapeutic tools and enables more efficient delivery to the tumor across biological barriers. The aim is complete removal of the tumor as even a miniscule amount of residual tumor can lead to fatal recurrence.

Immunotherapy is another promising approach and adjuvants have been found useful to enhance response to vaccines, and this approach has been evaluated inclinical trials of various vaccine therapies using autologous tumor antigens or tumor-associated/specific antigen peptide in patients with glioblastoma. Choice of an appropriate target and vaccination strategy combined with an immune modulator to increase the body's ability to mount an immune response against the tumor could lead to more durable responses in patients with glioblastoma. However, control of immune response is still inexact as overstimulation may have a destructive effect on normal tissues. Several clinical trials are currently being planned to test such immunomodulators. One clinical trial will test the combination of vaccination with immune checkpoint blockade There is a trend for immunotherapy to be integrated into the multimodal treatment including radiotherapy and chemotherapy for patients with glioblastoma as the actions of the individual treatment modalities may fortify each other (121). Future clinical trials of brain tumor vaccines may incorporate this strategy.

Because, there are limitations on extensive use and repetition of current treatments: surgery, chemotherapy and radiation, the ideal therapeutic agent should be one that can continue to act until the tumor is eradicated. Among novel biotechnologies, gene therapy is usually a one-time treatment and cannot be repeated for residual and recurrent tumor. Among genetically modified microorganisms, bacteria appear to be more promising than viruses. Genetically modifies bacteria that selectively destroy glioblastoma while sparing normal brain tissue can be more precise than any surgical tool and can be left in until the tumor destruction is complete. The bacteria can then be killed with an antibiotic.

Multimodal therapy of glioblastoma will be needed as no single method is adequate. Surgery will remain as an essential part of this multimodal approach unless a non-invasive or minimally invasive tool is developed to replace it.

There are interindividual difference in response to the growth of glioblastoma and response to treatment as well as variations in genomics of each tumor. Therefore, personalized approach to management of glioblastoma is important. Genetic and transcriptomic profiling of patient tumors by Next Gen Sequencing helps classify patients in to molecular subgroups for personalized approach of treatment. To this end, re-analysis of same genetic, and transcriptome data can help identify genetic or epigenetic alterations and discovery of new targets, that can be helpful in designing new drugs or nanoformulations that can be more efficient in drug delivery and efficacy (122).

## Author contributions

The author confirms being the sole contributor of this work and has approved it for publication.

### Conflict of interest statement

The author declares that the research was conducted in the absence of any commercial or financial relationships that could be construed as a potential conflict of interest.
